# Drying Behavior and Curcuminoids Changes in Turmeric Slices during Drying under Simulated Solar Radiation as Influenced by Different Transparent Cover Materials

**DOI:** 10.3390/foods11050696

**Published:** 2022-02-26

**Authors:** Nilobon Komonsing, Sebastian Reyer, Pramote Khuwijitjaru, Busarakorn Mahayothee, Joachim Müller

**Affiliations:** 1 Department of Food Technology, Faculty of Engineering and Industrial Technology, Silpakorn University, Nakhon Pathom 73000, Thailand; nilobon.komonsing@gmail.com (N.K.); khuwijitjaru_p@su.ac.th (P.K.); 2 Tropics and Subtropics Group, Institute of Agricultural Engineering, University of Hohenheim, 70599 Stuttgart, Germany; s.reyer@outlook.com (S.R.); joachim.mueller@uni-hohenheim.de (J.M.)

**Keywords:** *Curcuma longa*, herb, drying characteristic, ultraviolet, solar drying

## Abstract

Dried turmeric is used as a spice and traditional medicine. The common drying methods for turmeric (*Curcuma longa* L.) are sun drying and solar drying. In this study, turmeric slices with a thickness of 2 mm were dried at 40, 50, 60, and 70 °C in a laboratory hot-air dryer with a simulated solar radiation applied through transparent polycarbonate cover (UV impermeable) and PMMA cover (UV permeable). Air velocity and relative humidity of drying air were fixed at 1.0 M·s^−1^ and 25 g H_2_O kg^−1^ dry air, respectively. Light significantly increased the sample temperature under both covers. Page was the best model to predict the drying characteristics of turmeric slices. Drying rate correlated with the effective moisture diffusivity, which increased at higher temperature. The hue angle (*h*°) of turmeric was distinctly lower at 70 °C under both covers. The dried products were of intensive orange color. Curcumin, demethoxycurcumin, and total curcuminoids were affected by the cumulated thermal load (*CTL*). The lowest curcumin content was found at 40 °C under PMMA (highest *CTL*). The optimum drying condition was 70 °C under polycarbonate cover due to shorter drying time and better preservation of color and curcuminoids in the dried product.

## 1. Introduction

Solar dryers are using solar radiation as an energy source for drying agricultural products in a simple construction and have been extensively implemented in tropical and subtropical regions due to their affordability and cost-effectiveness [1,2,3]. Several types of solar dryers were developed to overcome drawbacks of direct sun drying, such as cabinet solar dryers [4,5], solar tunnel dryers [6,7], and greenhouse solar dryers [8,9]. These dryers provide temperatures higher than the ambient temperature, which results in faster drying compared to open-air sun drying. One of the most important components of a solar dryer is the transparent cover material which traps solar radiation and induces the greenhouse effect inside the solar dryer [8].

Various types of cover materials such as polyethylene, glass, polycarbonate, and poly(methyl methacrylate) sheets have been used for solar dryers [10,11,12,13,14]. Polyethylene is a widely used cover material for greenhouse dryers [12]. Although it is made of UV stabilized plastic, it lasts for only three to four months under strong UV radiation in tropical regions [15]. Clear glass transmits up to 90% of visible light and 72% of UV radiation [16], but its weak points are heavy weight and low shatter resistance. Polycarbonate has been used frequently because of its high resistance to impact [16] and effective UV radiation blocking [12]. The drying air temperature in a polycarbonate-covered greenhouse dryer reaches up to 65 °C in an environment with an ambient temperature of 35 °C [17]. Poly(methyl methacrylate) (PMMA), also known as plexiglass, transmits both UV and visible radiation with a transmittance of up to 92%. Both polycarbonate and PMMA can be used for many years. Cover materials influence the drying temperature and solar radiation inside the drying chamber, which may compromise the quality attributes and nutritional compositions of dried products [18,19].

Turmeric (*Curcuma longa* L.) is in the ginger family. The benefits of turmeric rhizomes are more than being used as a condiment, spice, coloring agent, or flavor boost because it is rich in curcuminoids (curcumin, demethoxycurcumin, and bisdemethoxycurcumin) and medicinal compounds [20]. Curcuminoids, orange-yellow pigments, are the most biologically active compounds in turmeric, which make up 2–6% of the rhizome’s dry mass [21]. The basic structures of curcuminoids are diarylheptanoid links between two aromatic rings [22], and they exhibit keto-enol tautomerism [23].

Light promotes the degradation of curcumin [24,25]. The chromophore group of curcumin strongly absorbs the visible light spectrum, making it susceptible to photochemical degradation which yields vanillin, vanillic acid, 4-vinylguaiacol, ferulic acid, and ferulic aldehyde [26]. The degradation causes fading of the yellow color of curcumin [25]. Curcumin in turmeric rhizomes is reduced by direct and indirect solar radiation. Raza et al. [27] reported lower curcumin contents of dried rhizomes from sun drying and solar tunnel drying than that from shade drying. Mahayothee et al. [28] also reported that drying in a solar greenhouse dryer covered with a polycarbonate sheet reduced curcumin contents in cassumunar ginger.

However, the study of cover materials on bioactive compounds of dried products is still limited. The main objective of this study was to investigate the effect of solar radiation exposure under two cover materials, one UV permeable and one UV impermeable, and different drying temperatures on drying characteristics, color, and curcuminoid contents of dried turmeric slices using a hot air dryer. Simulated solar radiation was employed to obtain consistent light intensity throughout the study, which is impossible in outdoor solar drying experiments.

## 2. Materials and Methods

### 2.1. Materials

Organic solvents used in the study were HPLC grade. Methanol (99.95%) and acetonitrile (99.9%) were from Geyer GmbH (Stuttgart, Germany) and abcr GmbH (Karlsruhe, Germany), respectively. Curcumin (purity ≥ 99.5%, CAS No. 458-37-7), demethoxycurcumin (purity ≥ 98%, CAS No. 22608-11-3), and bisdemethoxycurcumin (purity ≥ 98%, CAS No. 33171-05-0) were purchased from Sigma-Aldrich Chemie GmbH (Munich, Germany).

### 2.2. Turmeric Rhizomes

Turmeric (*C. longa* L.) rhizomes at the maturity of nine months were harvested from a plantation in Surat Thani province, Thailand. Rhizomes of uniform size, diameter (1.36 ± 0.18 cm) and weight (13.76 ± 3.70 g) were selected. Soil was brushed off the rhizomes before being packed in net bags. Thirty kilograms of the clean rhizomes were sent to the laboratory at the Institute of Agricultural Engineering, University of Hohenheim, and then kept in a refrigerator (Profiline GKv 6410-20, Liebherr, Biberach, Germany) at a temperature of 11.0 ± 0.4 °C and relative humidity of 90.7 ± 11.6%. The initial moisture content of the fresh rhizomes was 84.42 ± 0.70% (wet basis, w.b.). The stored rhizomes were washed under running tap water and allowed to drain at room temperature prior to drying experiments.

### 2.3. Simulated Light Exposure during Drying Experiments

#### 2.3.1. Experimental Set Up

**Figure 1:**
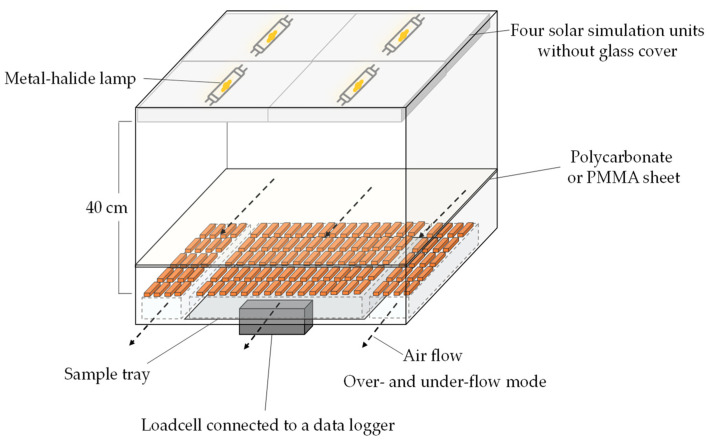
Depiction of the drying chamber and simulated solar light applied through the cover materials.

Drying experiments were conducted at 40, 50, 60, and 70 °C using the over- and under-flow chamber of a laboratory-made hot air dryer with precise controls for temperature and relative humidity. A detailed description of the drying system can be found in Argyropoulos et al. [29]. The air velocity was fixed at 0.5 m s^−1^ using a centrifugal blower. The specific humidity of the drying air was adjusted to 25 g water kg^−1^ air. Corresponding relative humidity at 40, 50, 60, and 70 °C was 50.8, 30.4, 18.8, and 12.0%, respectively. To evaluate the effect of light exposure during the drying experiments, a solar light generator was installed above the drying chamber (Figure 1). Four solar simulation units (SOL 500, Dr. Hönle AG, Starnberg, Germany) were used to simulate natural sunlight emitting at wavelengths of 280–2750 nm. Each unit has a supply voltage of 230 V/50 Hz and maximum power consumption of 430 W. The distance between the light bulbs and slice samples was fixed at 40 cm.

A 6 mm thick transparent double wall polycarbonate sheet with UV protection was used as a UV impermeable transparent cover (Twinlite^®^ Gen 2.0, PT Impack Pratama Industri Tbk, Jakarta, Indonesia). According to the manufacturer’s specification, transmittance is starting above a wavelength of 390 nm and the shortwave and longwave transmittance are 87 and 60%, respectively.

A 3 mm thick transparent PMMA sheet with high resistance to UV light was used as a UV permeable transparent cover (Plexiglas® GS clear 2548, Röhm GmbH, Darmstadt, Germany). According to the manufacturer’s specification, transmittance is starting above a wavelength of 250 nm and shortwave radiation transmittance is in the range of 90–92%. Either the polycarbonate or PMMA sheet was used to cover the drying chamber (Figure 1).

Prior to experiments, the dryer and light generator were warmed up to the required condition for an hour. Solar radiation above sample surfaces was measured using a solar radiation sensor with a spectral range of 360–1120 nm (SRS-200, Pace Scientific, Mooresville, NC, USA). Photon flux density under the polycarbonate and PMMA sheets was 1325 ± 125 µmol m^−2^ s^−1^ and 1557 ± 177 µmol m^−2^ s^−1^, respectively. UV radiation was measured using a UV radiometer with spectral range of 250–400 nm (SUVAB, GEOVES, Conegliano, Italy). The UV radiation under the polycarbonate and PMMA sheets were in the ranges of 0.6–0.9 W m^−2^ and 100–147 W m^−2^, respectively.

#### 2.3.2. Drying Experiment

The rhizomes were sliced using an adjustable hand slicer (mean thickness of 2.16 ± 0.28 mm). The slices were cut to a size of 10 mm × 40 mm using a handmade cutter. The samples (240 pieces, ca. 200 g) were placed on a perforated aluminum main tray in a single layer. An additional 100 pieces were placed on two side trays to create a homogeneous airflow in the drying chamber. The product temperature was measured by inserting thin mantle thermocouples in two pieces when the samples were placed in the drying chamber. The product temperature was recorded during the process as well. The main tray was automatically weighed every 15 min using a load cell (Flintec, type 123 PC6, Västerås, Sweden) over the course of drying at 40 and 50 °C and every 10 min at 60 and 70 °C. Once the product mass approached a constant value, samples from the side trays were randomly taken to measure the water activity (*a*_w_) using a water activity meter (HP23 AW-A, Rotronic, Bassersdorf, Switzerland) in order to confirm that the *a*_w_ value of the product had reached 0.360, which corresponds to the moisture content of the FAO recommendation [30]. The dried product was kept in a jar for 24 h to reach equilibrium prior to chemical analysis. Initial and final moisture contents were determined using a vacuum dryer (VT 6060 P, Thermo Scientific, Waltham, MA, USA) at 50 °C and 2 kPa for 12 h. The drying experiment was performed in duplicate.

### 2.4. Evaluation of Drying Characteristics

Moisture contents at different times on both a wet basis and a dry basis were calculated. The initial mass was obtained by measuring the mass of turmeric slices before drying. The dry solid mass of the sample was calculated from final moisture content of dried products.

The drying rate (*DR*) (g water g^−1^ dry solid h^−1^ m^−2^) was then calculated using Equation (1):

(1) $$DR=\frac{\Delta M_{db}}{\Delta t\cdot A}=\frac{M_{1,\;db\;}-\;M_{2,\;db}}{t_{2}-t_{1}}\cdot \frac{1}{A}$$

where *M*_1,*db*_ and *M*_2,*db*_ are moisture contents (g water g^−1^ dry solid) at times *t*_1_ and *t*_2_ (h), respectively and *A* is surface area (m^2^).

The moisture ratio (*MR*) of turmeric slices during the drying experiment was calculated using Equation (2):

(2) $$MR=\frac{M_{t}-M_{e}}{M_{0}-M_{e}}$$

where *M*_0_ and *M*_t_ are the initial moisture content and the moisture content at a specific time (g water g^−1^ dry solid), respectively. The equilibrium moisture content (*M*_eq_) was defined as the final moisture content (g water g^−1^ dry solid) for each drying condition.

Effective moisture diffusivity (*D*_eff_) is used to describe drying characteristics of foodstuffs by the diffusion phenomenon. Due to the slab shape of the sliced turmeric, Fick’s second law was used to determine *D*_eff_ by Equation (3) [31]:

(3) $$MR=\frac{8}{\pi^{2}}\cdot \exp\left(\frac{-\pi^{2}\cdot D_{\mathrm{eff}}\cdot t}{4\cdot h^{2}}\right)$$

where *D*_eff_ is the effective moisture diffusivity (mm^2^ h^−1^) and h is the half thickness of the slab (mm). Equation (3) can be written in a logarithmic form as Equation (4) [31]:

(4) $$\ln MR=\ln\frac{8}{\pi^{2}}-\frac{\pi^{2}\cdot D_{\mathrm{eff}}}{4\cdot h^{2}}\cdot t$$

The slope value was obtained from the plot of ln *MR* versus time *t* (Equation (4)). *D*_eff_ was calculated using Equation (5):

(5) $$D_{\mathrm{eff}}=\;\mathrm{slope}\;\cdot \frac{-4\cdot h^{2}}{\pi^{2}}$$

Activation energy (*E*_a_) was estimated to describe the relation between the *D*_eff_ and temperature by an Arrhenius-type equation as shown in Equation (6) [32]:

(6) $$D_{\mathrm{eff}}=D_{0\;}\exp\;\left(\frac{-E_{a}}{RT}\right)$$

where *D*_0_ is the pre-exponential factor (mm^2^ h^−1^), *E*_a_ is the activation energy for the moisture diffusion (kJ mol^−1^), *R* is the ideal gas constant (kJ mol^−1^ K), and *T* is the drying temperature (K). Equation (6) can be written in a logarithmic form as Equation (7):

(7) $$\ln\;D_{\mathrm{eff}}=\ln\;D_{0\;}-\frac{E_{a}}{RT}$$

*E*_a_ was obtained from the plot of ln *D*_eff_ versus 1/*T*. The slope of the fitted straight line is −*E*_a_/*R*.

### 2.5. Thin Layer Drying Models

The thin-layer drying equation is important for drying kinetics analysis. It gives some understanding of the water transportation process during drying. *MR* from experimental data were fitted with various thin-layer drying models. The Lewis model (Equation (8)) is derived from Newton’s law of cooling [33]:

(8) MR=exp(−k·t)

where *k* is the drying constant (h^−1^) and *t* is the drying time (h).

Page model [34] (Equation (9)) and Modified Page model [35] (Equation (10)) were modified from Lewis to obtain more accuracy by adding a parameter *n* as a power of *t* and of (−*kt*), respectively:

(9) $$MR=\exp\left(-k\cdot t^{n}\right)$$

(10) $$MR=\exp{\left(-k\cdot t\right)}^{n}$$

Henderson and Pabis model (Equation (11)) was derived from the model of Fick’s second law of diffusion by adding a shape parameter *a*. The Logarithmic model (Equation (12)) was modified from Henderson and Pabis model with a parameter *b* [35].

(11) MR=a·exp(−k·t)

(12) MR=a·exp(−k·t)+b

Midilli and Kucuk model [36] (Equation (13)) was obtained by adding both a parameter *n* as a power of *t* and another term with a parameter *b* (h^−1^) multiplied with *t* to the Henderson and Pabis model:

(13) $$MR=a\cdot \exp\left(-k\cdot t^{n}\right)+b\cdot t$$

The models were evaluated by the coefficient of determination (*R*^2^), the root mean square error (*RMSE*), and the Akaike information criterion (*AIC*) [37] as given in Equations (14)–(16). High *R*^2^, low *RMSE* and *AIC* are preferable.

(14) $$R^{2}=\frac{Residual\;sum\;of\;square}{Total\;sum\;of\;square}$$

(15) $$RMSE=\sqrt{\frac{\sum_{i=1}^{n}{\left(MR_{i,exp}-MR_{i,pre}\right)}^{2}}{n}}$$

(16) AIC=− 2·ln(L)+2·k

where *MR_exp_* is the experimental moisture ratio, *MR_pre_* is the moisture ratio predicted by the thin layer models, *n* is the number of observations, *L* is the maximum value of the likelihood for the model, and *k* is the number of parameters of the model. All models were fitted using a non-linear regression analysis in R version 3.5.1 [38].

To evaluate the effect of time and temperature during the drying process, the thermal energy received by the sample was estimated using the cumulated thermal load (*CTL,* K h) according to Jödicke et al. [39] (Equation (17)):

(17) $$CTL=\underset{n=0}{\sum }\frac{\left(T_{n}-T_{o}\right)+\left(T_{n+1}-T_{o}\right)}{2}\cdot \left(t_{n+1}-t_{n}\right)$$

where *T_n_* is the product temperature (K) at measurement event *n*, *T*_0_ is the initial product temperature (33.8 °C), and *t_n_* is the drying time (h) at measurement event *n*.

### 2.6. Color Measurement

Chroma meter (CR-400, Minolta, Tokyo, Japan) was used to measure the color of fresh turmeric in the CIE L* a* b* color space for each drying condition from 30 fresh turmeric slices (two positions per piece). The dried sample was ground into powder using a knife mill (Grindomix GM 200, Retsch, Haan, Germany) at 10,000 min^−1^ for 30 s. The process was paused for an interval time of 10 s after each 10 s of grinding. The ground powder was passed through a sieve No. 35 (pore size 500 µm). The sample was filled in a CR-A50 granular material attachment before color measurements. Five replications were measured. Chroma (C*), hue angle (*h*°), and total color change (Δ*Ε*) were calculated using Equations (18)–(20), respectively:

(18) $$C^{*}=\sqrt{a^{*}{}^{2}+b^{*}{}^{2}}$$

(19) $$h^{*}=\tan^{-1}\left(\frac{b^{*}}{a^{*}}\right)$$

(20) $$\Delta E=\sqrt{\Delta L^{{*}^{2}}+\Delta a^{*}{}^{2}+\Delta b^{*}{}^{2}}$$

where Δ refers to the difference of each parameter between fresh turmeric and turmeric powder.

### 2.7. Methanolic Extraction

Three grams of mashed fresh turmeric or 1.5 g of powder was extracted in 20 mL of methanol using an ultrasonic bath (Transsonic T-780/h, Elma, Stuttgart, Germany) for 30 min. The water temperature was controlled to be lower than 35 °C. The mixture was centrifuged at 11,530× *g* for 15 min at 4 °C (Z326K, Hermle, Gosheim, Germany). The supernatant was collected in a 100 mL volumetric flask and the residue was re-extracted another two times with 20 mL of methanol each. The pooled supernatant was adjusted to 100 mL with methanol and then filtered through a 0.45 µm nylon syringe filter before the analysis. The extractions were performed in triplicate.

### 2.8. Chromatographic Analysis of Curcuminoids

The curcumin, demethoxycurcumin, and bisdemethoxycurcumin contents were immediately determined using a Shimadzu HPLC system (Kyoto, Japan) consisting of a 2IL-20AC HT autosampler, DGU-20A5R degasser, LC-20AT pump, SPD-M20A diode array detector, and CTO-20A column heater. Chromatographic separation was conducted using a Luna C18 column (250 × 4.6 mm i.d.; 5 µm; Phenomenex, Torrance, CA, USA) operated at 30 °C. The autosampler temperature was 4 °C. The mobile phases A and B were acetonitrile and 1% acetic acid solution, respectively. The gradient mode elution was carried out as follows: a linear decrease from 60 to 50% B in 30 min, 50 to 35% B in 5 min, 35 to 30% B in 5 min and maintained at 30% for 8 min, 30–0% B in 3 min, and 0–60% B in 7 min at a flow rate of 1.0 mL min^−1^. The injection volume was 10 µL. Curcuminoid peaks were monitored at 425 nm. Standard curcumin, demethoxycurcumin, and bisdemethoxycurcumin in methanol at concentration range of 1.0–75.0 mg L^−1^ were used for preparation of standard curves. The contents of each component were calculated in mg g^−1^ dry solid. Total curcuminoids content was the sum of the contents of curcumin, demethoxycurcumin, and bisdemethoxycurcumin.

### 2.9. Statistical Analysis

Analysis of variance (ANOVA) and Duncan’s multiple range test were performed to evaluate the difference of each response variable. The results were assessed at a probability level of 0.05. A statistical analysis was performed using SPSS Statistics 17.0 (IBM, Chicago, IL, USA).

## 3. Results and Discussion

### 3.1. Drying Characteristics

Turmeric slices with an initial moisture content of 84.24 ± 0.70% (w.b.) were dried to final moisture content and water activity in the ranges of 6.23–8.54% (w.b.) and 0.331–0.365 (Table 1) which were in accordance with the requirements for dried turmeric [30].

**Figure 2:**
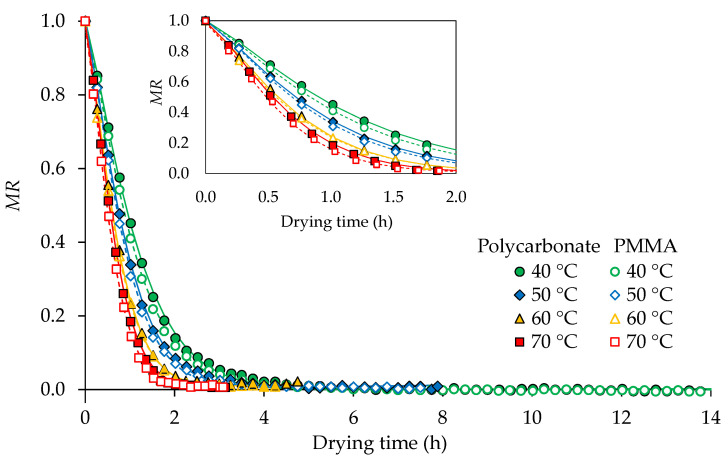
Drying curves of turmeric slices as affected by temperature and different cover materials. The lines show the predicted moisture ratio (*MR*) from the Page model using generalized *k* values from Equations (21) and (22).

**Figure 3:**
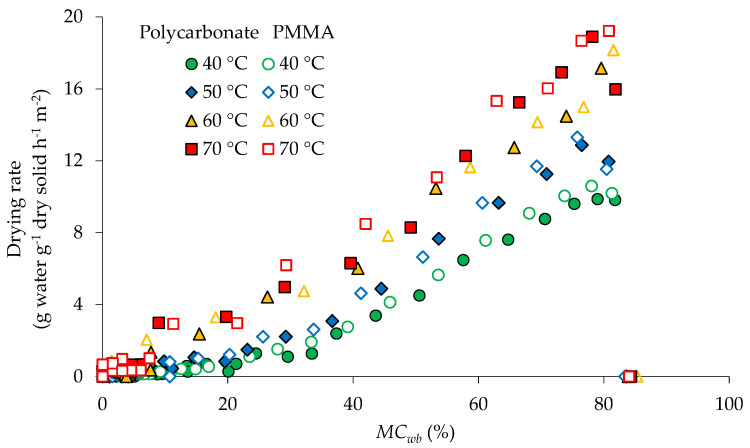
Drying rate of turmeric slices as affected by temperature and different cover materials. *MC*_wb_: Moisture content (wet basis).

The drying curves at 40, 50, 60, and 70 °C for both polycarbonate and PMMA covers are presented in Figure 2. MR reduced rapidly at the beginning of the drying process and the highest drying rates were also observed in this period (Figure 3). 

**Figure 4:**
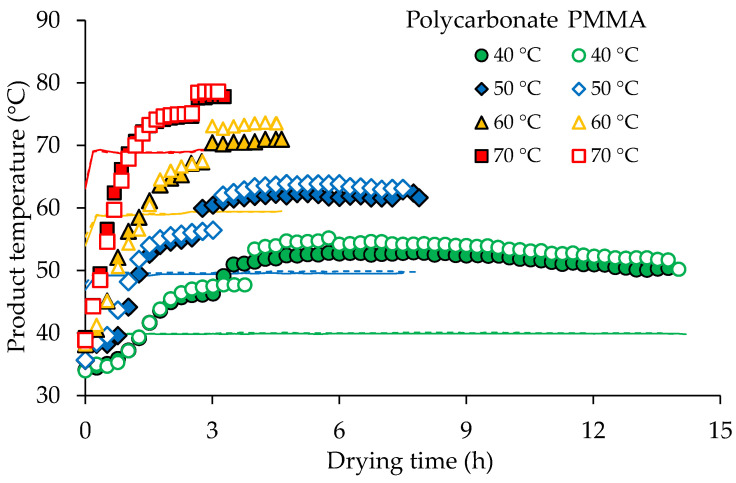
Product temperature under polycarbonate and PMMA cover; lines and dotted lines show the drying air temperature in the drying chamber under polycarbonate and PMMA cover, respectively.

The MR reduction was, on a small scale, faster under the polycarbonate cover due to the slightly higher product temperatures (Figure 4). The drying time required to reach the constant moisture content was shorter at higher temperatures (Table 1). It ranged between 3.17 h (at 70 °C) and 14.22 h (at 40 °C). The *CTL* values show the amount of heat assimilated by the samples throughout the drying process (Table 1). Because *CTL* reflects the combined effect of time and temperature, longer drying times can result in higher CTL values even at lower drying temperatures. At 40 and 50 °C, drying under PMMA cover showed higher *CTL* values than polycarbonate cover corresponding to higher product temperatures (Figure 4). However, cover materials did not significantly affect the *CTL* values at 60 and 70 °C. The lowest *CTL* values were found at 70 °C.

Figure 4 shows average temperatures inside the product during drying. The initial temperature of fresh turmeric slices was 33.8 °C. At the beginning of the drying process, the product temperatures were lower than the drying air temperatures and rose rapidly due to heat transfer from the hot air to the samples. The drying rate increased swiftly to its maximum value as the product temperature increased (Figure 3). The initial moisture content of fresh turmeric slices was reduced from approximately 84% to 81% (w.b.) within 15 min at 40 and 50 °C and 10 min at 60 and 70 °C for both covers. This phase is known as the warm-up period. Drying at higher temperatures led to a shorter warm-up period. The rates of drying were higher at higher temperatures, which were caused by the higher energy available to vaporize free water off the turmeric surfaces. The falling-rate period started when the moisture content of the samples reached a critical moisture content of approximately 80% (w.b.) (Figure 3). In this period, the free surface water was insufficient for continuous evaporation, resulting in case hardening and shrinkage of the sample surface. This also led to an increase in surface temperature and a decrease in mass transfer driving force. Figure 4 indicates that the product temperatures rose to the set drying air temperatures in an hour and exceeded these by approximately 10 °C within 3 h for both cover materials. This temperature difference could be explained by the absorption of solar radiation by the samples which was converted into thermal energy. After 3 h of drying, the product temperature under the PMMA cover was slightly higher (≈1 °C) than under the polycarbonate cover. This can be attributed to the higher light transmittance of the PMMA cover compared to polycarbonate.

The thin layer models (Lewis, Page, Modified Page, Henderson and Pabis, and Midilli and Kucuk) were fitted to the *MR* from the initial time to the beginning of constant weight (at 5.00, 4.00, 2.75, 2.00 and 4.75, 3.75, 2.50, 2.00 h for 40, 50, 60, and 70 °C under polycarbonate and PMMA covers, respectively) to avoid errors from long constant weight data in parameter modeling. The Page, Modified Page, and Midilli and Kucuk models yielded the highest *R*^2^, lowest *RMSE*, and *AIC* values, as shown in Appendix A (Table A1). Therefore, the Page model (Equation (9)), which is simple and widely used, was considered to be the best model for drying turmeric slices. The modeling results showed that *k* increased significantly with drying temperature (*p* < 0.05). However, no significant differences of *k* between polycarbonate and PMMA covers were found. Therefore, it can be concluded that temperature has the largest impact on the drying rate. Temperature affects the drying rate by the acceleration of evaporation, diffusivity, and heat transfer during drying and the subsequent increase in water migration from inside the product to the drying air [40].

The *k* values from the Page model changed with the drying temperature. They could be well described by the exponential equation with the high *R*^2^ values. The generalized equations describing the *k* values as a function of drying temperature (*T*_C_, °C) for the drying under the polycarbonate and PMMA sheets are shown in Equations (21) and (22).

(21) $$\mathrm{Polycarbonate}\;(R^{2}=0.9734):\;k=0.2766\cdot \exp\left(0.0262\cdot T_{C}\right)$$

(22) $$\mathrm{PMMA}\;(R^{2}=0.9907):\;k=0.3256\cdot \exp\left(0.0250\cdot T_{C}\right)$$

On the other hand, the *n* values at different drying temperatures were almost constant and the average values of 1.2742 and 1.2629 were obtained for polycarbonate and PMMA covers, respectively. The calculated *k* values from the generalized equations (Equations (21) and (22)) and the average *n* values were used to predict changes in the moisture ratio during drying (Lines in Figure 2). It was found that the model could well explain the drying behavior of turmeric slices at different temperatures with a high *R*^2^ (0.9187–0.9759) and low *RMSE* (0.0052–0.0176).

**Figure 5:**
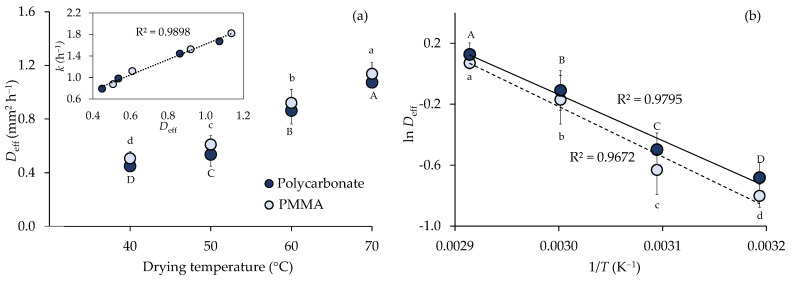
Effective moisture diffusivity (*D*_eff_) of turmeric slices as affected by temperature and different cover materials (**a**) and Arrhenius relationship between ln *D*_eff_ and reciprocal temperature (1/*T*) (**b**). Different letters indicate significantly different (*D*_eff_) at *p* < 0.05. Drying conditions are indicated by 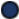 polycarbonate and 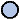 PMMA. *k*: drying rate.

The moisture movement in the sample during the falling-rate period is described by molecular diffusion [41]. Figure 5a shows that the effective moisture diffusivity (*D*_eff_) values of turmeric slices during drying significantly increased with the rising temperature (*p* < 0.05). *D*_eff_ was linearly proportional to the drying rate *k* (from the Page equation) with *R*^2^ = 0.9898 (Figure 5a inset). The obtained *D*_eff_ values were 0.45, 0.54, 0.86, and 1.07 mm^2^ h^−1^ at 40, 50, 60, and 70 °C, respectively for polycarbonate covering and 0.51, 0.61, 0.92, and 1.14 mm^2^ h^−1^, respectively for PMMA covering. *D*_eff_ was obviously affected by drying temperature. However, the statistical analysis showed a non-significant difference of *D*_eff_ between polycarbonate and PMMA covers at the same temperature. Figure 5b illustrates the effect of temperature under different cover materials by the Arrhenius-type relationship. Activation energies were 21.55 ± 2.75 kJ mol^−1^ for polycarbonate cover and 21.20 ± 1.72 kJ mol^−1^ for PMMA cover.

### 3.2. Appearance and Color Measurement

**Figure 6:**
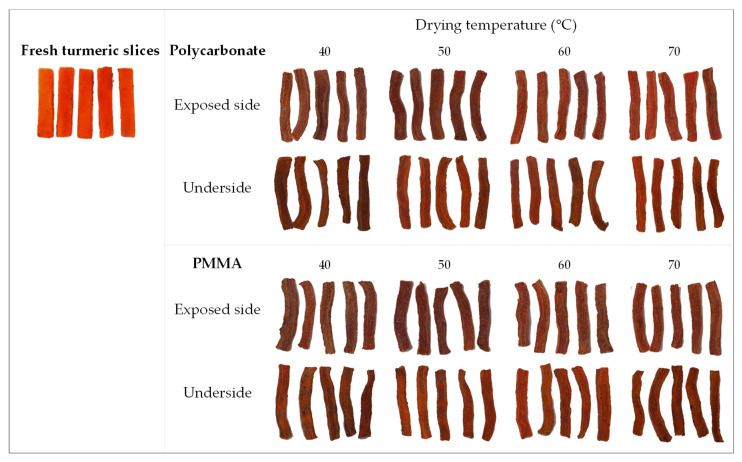
Appearance of fresh and dried turmeric slices on both exposure side and underside after drying under polycarbonate and PMMA covers, respectively.

Figure 6 shows appearances of fresh and dried turmeric slices obtained from various drying conditions. The fresh turmeric was of intense orange color. Drying temperatures and different covers affected the appearance of the dried products. For both covers, higher temperature resulted in less color fading on the surfaces due to shorter light exposure. The most brownish surfaces were found from polycarbonate cover at 40 and 50 °C and PMMA cover at 40 °C. The underside of the products at 50 and 60 °C under both covers was somewhat brighter than the exposed side. The dried product at 70 °C under polycarbonate cover showed the most intense orange on both sides, while the slices dried under PMMA cover at the same temperature were darker. This difference could be the effect of higher UV intensity under PMMA.

Table 2 shows the color values of turmeric powder from various conditions. Drying temperature and covers significantly affected a* values of the powder (*p* < 0.05), which signifies the redness of turmeric flesh. The a* value was lowest at 40 °C under polycarbonate and PMMA and at 70 °C under PMMA. This corresponded with the appearance of the dried products. Pal et al. [42] showed that the a* value was an important indicator of the quality of turmeric rhizomes. The L*, b*, and C* values of the dried powder were lowest at 70 °C under both covers. The powder from these conditions was of dark orange color.

### 3.3. Degradation of Curcuminoids

The orange color of turmeric is due to the curcuminoid pigments. Fresh turmeric slices used in this study had a total curcuminoids content of 8.06 ± 0.11 g 100 g^−1^ fresh sample. Three curcuminoids were identified by HPLC-DAD as curcumin (64.75%), demethoxycurcumin (16.65%), and bisdemethoxycurcumin (18.60%). Wichitnithad et al. [43] reported that the curcuminoids in most commercial turmeric extracts consisted of 60–80% curcumin, 15–30% demethoxycurcumin, and 2–6% bisdemethoxycurcumin. The curcumin, demethoxycurcumin, and bisdemethoxycurcumin contents of the fresh samples were in the ranges of 197.03–224.95, 51.31–56.71, and 57.09–64.13 mg g^−1^ dry solid, respectively (Table 3). It was found that the drying process reduced curcumin, demethoxycurcumin, and total curcuminoids contents under all conditions, except at 70 °C under polycarbonate cover. Curcumin contents of the turmeric powder were in the range of 182.50–213.72 mg g^−1^ dry solid. The bisdemethoxycurcumin contents at 40 °C under polycarbonate and PMMA covers were not significantly different from the fresh sample. At 50, 60, and 70 °C the bisdemethoxycurcumin contents increased under both covers.

**Figure 7:**
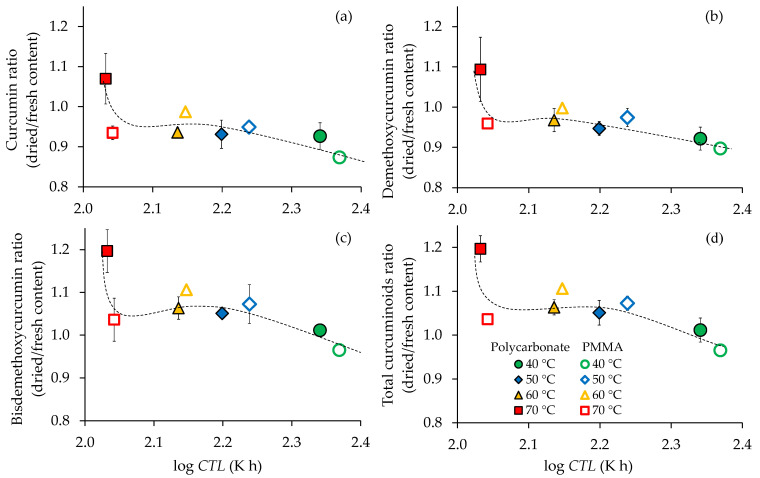
Degradation of curcuminoids in function of cumulated thermal load (*CTL*); (**a**) curcumin, (**b**) demethoxycurcumin, (**c**) bisdemethoxycurcumin, and (**d**) total curcuminoids.

The ratios between each curcuminoid in turmeric powder and fresh turmeric are plotted against the logarithm of the cumulated thermal load (log *CTL*) values (Figure 7) to illustrate the combined time-temperature effect on curcuminoid degradation during the drying under different conditions. A ratio above unity indicates an increase in curcuminoids in the obtained turmeric powder compared to fresh turmeric, while a ratio lower than unity indicates the degradation of curcuminoids. All curcuminoids ratios rapidly decreased when the log *CTL* increased from 2.02 to 2.37 and then tended to gradually decrease afterwards (Figure 7). The highest degradation of all curcuminoids was found at 40 °C under PMMA. This is likely because of the long drying time and exposure to light (14.22 h). The results were in agreement with the study of Komonsing et al. [44] which found that curcuminoids in turmeric slices were degraded by temperature and light under the long time drying, while the curcuminoid contents of the products obtained from drying in the dark were higher than those of the fresh sample. At 40 °C, the drying under polycarbonate cover, which can protect UV radiation, showed less degradation of curcuminoids. Few studies observed that the photodegradation of curcuminoids was strongly caused by exposure to UV radiation [45,46]. Degradation of bisdemethoxycurcumin was found only at 40 °C under PMMA cover. This suggests a higher stability of bisdemethoxycurcumin towards solar radiation compared to curcumin, which was the most sensitive, and demethoxycurcumin.

Figure 7 also suggests that the type of cover material (polycarbonate or PMMA) did not significantly (*p* > 0.05) affect the curcuminoids ratio at 50 and 60 °C. However, a significant difference was found at 70 °C (lowest log *CTL*). Drying under PMMA led to the degradation of curcumin (0.93) and demethoxycurcumin (0.91) in the powder, which might be due to the combination of UV radiation and high temperature accelerating the degradation of these curcuminoids. By contrast, the powder from polycarbonate showed higher curcumin and demethoxycurcumin contents after drying. The increase in curcuminoids contents at 70 °C under polycarbonate might be due to the lowest log *CTL* together with the absence of UV radiation. Souza et al. [47] found that the combination of light and hot air was more detrimental to curcumin than only one of both factors. Lee et al. [25] reported that curcumin is decolorized when exposed to UV light. The structure of curcuminoids, which are chromophores, can absorb UV radiation, which results in auxochromes exhibiting brown color [48]. UV induced about 50% of fading of the yellow color of curcumin solution within 8 h [25]. However, the photodegradation of curcuminoids has not been well elucidated. It is likely that curcumin acts as a photosensitizer and decomposes in the process [49]. The degraded products might be ferulic aldehyde, ferulic acid, 4- vinylguaiacol, vanillin, and vanillic acid. The result agreed with Rodríguez-Ramírez et al. [12] who reported that cover materials (polycarbonate and polyethylene) influenced the drying temperature and UV radiation inside the solar drying chamber. They found that dried strawberries obtained from polyethylene cover (UV permeable) showed lower total phenolic contents due to higher temperature compared to polycarbonate (UV impermeable) cover. The degradation of anthocyanin contents was also lower in the absence of UV.

## 4. Conclusions

This study was conducted to imitate solar drying under transparent polycarbonate cover (UV impermeable) and PMMA covers (UV permeable). Light increased product temperature, which increased in drying rates and effective moisture diffusivity. The combined time-temperature effect was presented as cumulated thermal load (*CTL*), which could be used to describe the degradation of curcuminoids. In addition, UV radiation transmitted through PMMA increased the degradation of curcuminoids. In conclusion, the best quality of dried products in terms of color and curcuminoids contents were achieved by drying at 70 °C under polycarbonate cover. Drying under these conditions resulted in a shorter drying time without any negative effect on the contents of the curcuminoids. This knowledge can be applied for the optimization of the drying process for turmeric slices in a solar dryer.

## Figures and Tables

**Table 1:** Drying time, final moisture content (MC), and water activity (a_w_) of dried products, and cumulated thermal load (*CTL*) during drying at various drying conditions.

Temperature (°C)	Drying Time (h)	Cover Materials	MC (% w.b.) ^ns^	a_w_ ^ns^	*CTL* (K h)
40	14.22	Polycarbonate	8.06 ± 1.84	0.331 ± 0.029	219.25 ± 5.21 ^b^
		PMMA	8.54 ± 0.40	0.365 ± 0.013	233.89 ± 10.51 ^a^
50	7.50	Polycarbonate	7.30 ± 1.23	0.363 ± 0.016	158.13 ± 7.92 ^d^
		PMMA	6.53 ± 1.24	0.350 ± 0.047	173.20 ± 4.00 ^c^
60	4.75	Polycarbonate	7.08 ± 0.81	0.331 ± 0.017	136.68 ± 1.65 ^e^
		PMMA	7.13 ± 1.47	0.347 ± 0.019	140.33 ± 1.17 ^e^
70	3.17	Polycarbonate	6.23 ± 0.94	0.336 ± 0.004	107.71 ± 1.56 ^f^
		PMMA	6.51 ± 0.18	0.334 ± 0.021	110.23 ± 1.21 ^f^

Values are given as the mean ± standard deviation. Different superscript letters in columns indicate significant differences (*p* < 0.05). ns: not significant.

**Table 2:** Appearance and color values of the fresh sample and dried turmeric powder obtained from various drying conditions.

		Drying Temperature (°C)							
Color Values	Fresh Sample	40		50		60		70	
		Polycarbonate	PMMA	Polycarbonate	PMMA	Polycarbonate	PMMA	Polycarbonate	PMMA
	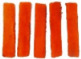	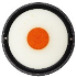	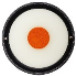	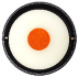	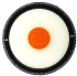	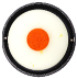	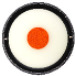	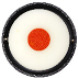	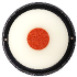
L*	53.37 ± 0.46	54.59 ± 0.12 ^a^	51.36 ± 2.86 ^a^	54.09 ± 1.91 ^a^	53.10 ± 0.16 ^a^	54.24 ± 0.37 ^a^	52.02 ± 4.37 ^a^	49.67 ± 0.37 ^b^	47.80 ± 1.08 ^b^
a*	38.34 ± 1.00	26.32 ± 0.76 ^bcd^	25.85 ± 1.39 ^cd^	27.99 ± 0.08 ^abc^	27.37 ± 0.33 ^abc^	28.46 ± 1.07 ^ab^	26.46 ± 0.10 ^abc^	28.63 ± 0.44 ^a^	24.18 ± 1.55 ^d^
b*	47.47 ± 1.58	34.43 ± 0.04 ^a^	28.59 ± 4.68 ^ab^	34.41 ± 3.32 ^a^	32.27 ± 0.08 ^a^	34.29 ± 0.17 ^a^	30.60 ± 5.59 ^a^	26.99 ± 0.53 ^ab^	22.94 ± 2.04 ^b^
C*	61.09 ± 1.51	43.34 ± 0.42 ^a^	38.57 ± 4.40 ^ab^	44.39 ± 2.52 ^a^	42.32 ± 0.15 ^a^	44.58 ± 0.56 ^a^	40.54 ± 4.16 ^a^	39.35 ± 0.64 ^ab^	33.34 ± 2.48 ^b^
*h*°	50.02 ± 1.04	52.61 ± 0.84 ^a^	47.71 ± 3.15 ^ab^	50.80 ± 2.79 ^a^	49.70 ± 0.40 ^a^	50.31 ± 1.20 ^a^	48.87 ± 5.30 ^ab^	43.31 ± 0.36 ^b^	43.45 ± 1.07 ^b^
Δ*E*	-	16.46 ± 2.96 ^c^	22.86 ± 2.98 ^abc^	16.56 ± 1.92 ^c^	18.59 ± 2.09 ^bc^	18.08 ± 0.03 ^bc^	21.25 ± 4.47 ^bc^	23.68 ± 2.50 ^ab^	27.94 ± 1.01 ^a^

Values are given as the mean ± standard deviation. Different superscript letters indicate significant differences (*p* < 0.05). *h*°: hue angle, Δ*E*: Total color change.

**Table 3:** Curcumin, demethoxycurcumin, and bisdemethoxycurcumin of turmeric slices obtained from various drying conditions.

Temperature (°C)	Cover Materials	Curcumin		Demethoxycurcumin		Bisdemethoxycurcumin		Total Curcuminoid Content	
		(mg g^−1^ Dry Solid)		(mg g^−1^ Dry Solid)		(mg g^−1^ Dry Solid)		(mg g^−1^ Dry Solid)	
		Fresh	Dried	Fresh	Dried	Fresh	Dried	Fresh	Dried
40	Polycarbonate	197.03 ± 3.09 ^a^	182.50 ± 3.67 ^b^	51.31 ± 1.08 ^a^	47.27 ± 0.48 ^b^	57.09 ± 0.39 ^a^	57.75 ± 0.03 ^a^	305.44 ± 4.56 ^a^	287.52 ± 4.12 ^b^
	PMMA	211.97 ± 3.20 ^a^	184.99 ± 2.89 ^b^	54.67 ± 0.03 ^a^	49.06 ± 0.71 ^b^	62.56 ± 1.57 ^a^	60.37 ± 1.61 ^a^	329.03 ± 1.60 ^a^	294.42 ± 0.58 ^b^
50	Polycarbonate	217.13 ± 4.00 ^a^	202.11 ± 3.92 ^a^	56.48 ± 0.19 ^a^	53.47 ± 1.16 ^a^	62.25 ± 1.64 ^a^	65.42 ± 2.34 ^a^	335.86 ± 2.16 ^a^	321.00 ± 7.42 ^a^
	PMMA	206.10 ± 5.48 ^a^	195.62 ± 6.44 ^a^	53.13 ± 1.46 ^a^	51.77 ± 2.64 ^a^	59.41 ± 1.82 ^a^	63.76 ± 4.64 ^a^	318.64 ± 8.75 ^a^	311.16 ± 13.72 ^a^
60	Polycarbonate	224.95 ± 3.78 ^a^	210.48 ± 0.66 ^b^	56.71 ± 1.93 ^a^	54.85 ± 0.25 ^a^	64.13 ± 3.02 ^a^	68.15 ± 1.51 ^a^	345.79 ± 8.72 ^a^	333.49 ± 2.24 ^a^
	PMMA	202.76 ± 1.82 ^a^	200.17 ± 3.45 ^a^	52.98 ± 1.34 ^a^	52.86 ± 1.33 ^a^	57.76 ± 3.63 ^a^	63.87 ± 3.81 ^a^	313.50 ± 6.79 ^a^	316.90 ± 8.59 ^a^
70	Polycarbonate	200.22 ± 14.42 ^a^	213.72 ± 2.84 ^a^	52.09 ± 3.56 ^a^	56.97 ± 1.00 ^a^	59.44 ± 5.81 ^a^	70.69 ± 2.17 ^a^	311.75 ± 23.78 ^a^	341.20 ± 0.33 ^a^
	PMMA	216.86 ± 5.95 ^a^	202.62 ± 1.88 ^a^	55.83 ± 1.87 ^a^	53.55 ± 2.24 ^a^	63.28 ± 0.27 ^a^	65.56 ± 3.46 ^a^	335.96 ± 8.09 ^a^	321.71 ± 7.59 ^a^

Values are given as the mean ± standard deviation. Different superscript letters indicate significant differences between the fresh and dried sample for each compound (*p* < 0.05).

## Data Availability

The data presented in this study are available on request to the corresponding author.

## Appendix A. Supplementary Data

**Table A1:** Drying constants and statistical results for drying of turmeric slices at different conditions.

Temperature (°C)	Cover Materials	Newton				Page					Modified Page					Henderson and Pabis					Midilli and Kucuk						
		*k*	*R* ^2^	*RMSE*	*AIC*	*k*	*n*	*R* ^2^	*RMSE*	*AIC*	*k*	*n*	*R* ^2^	*RMSE*	*AIC*	*k*	*a*	*R* ^2^	*RMSE*	*AIC*	*a*	*k*	*n*	*b*	*R* ^2^	*RMSE*	*AIC*
40	Polycarbonate	0.8547	0.9899	0.0295	−84.34	0.7930	1.2225	0.9986	0.0110	−123.82	0.8272	1.2225	0.9986	0.0110	−123.82	0.9002	1.0576	0.9931	0.0245	−90.14	0.9992	0.8021	1.2700	0.0037	0.9994	0.0074	−136.32
	PMMA	0.9344	0.9888	0.0306	−83.90	0.8778	1.2462	0.9987	0.0080	−130.62	0.9007	1.2462	0.9987	0.0080	−130.62	0.9842	1.0587	0.9921	0.0256	−78.74	0.9994	0.8874	1.2803	0.0030	0.9995	0.0049	−146.10
50	Polycarbonate	1.0205	0.9888	0.0320	−64.80	0.9823	1.2446	0.9987	0.0108	−101.84	0.9858	1.2446	0.9987	0.0108	−101.84	1.0731	1.0566	0.9920	0.0271	−68.48	1.0012	1.0076	1.2946	0.0051	0.9996	0.0060	−117.86
	PMMA	1.1373	0.9882	0.0331	−59.70	1.1218	1.2423	0.9992	0.0086	−100.88	1.0948	1.2679	0.9992	0.0086	−100.88	1.1946	1.0561	0.9914	0.0284	−62.60	1.0018	1.1488	1.3039	0.0042	0.9998	0.0045	−117.38
60	Polycarbonate	1.3935	0.9884	0.0340	−43.50	1.4431	1.2703	0.9996	0.0065	−80.88	1.3473	1.2703	0.9996	0.0065	−80.88	1.4463	1.0434	0.9905	0.0308	−43.10	0.9965	1.4594	1.2940	0.0026	0.9997	0.0057	−79.90
	PMMA	1.4653	0.9885	0.0343	−38.58	1.5248	1.2545	0.9990	0.0103	−63.46	1.3998	1.2545	0.9990	0.0103	−63.46	1.5122	1.0370	0.9901	0.0319	−38.96	0.9956	1.4715	1.2233	−0.0063	0.9994	0.0081	−64.80
70	Polycarbonate	1.5337	0.9788	0.0468	−41.98	1.6728	1.3594	0.9997	0.0054	−100.26	1.4601	1.3593	0.9997	0.0054	−100.26	1.6294	1.0706	0.9841	0.0405	−44.04	0.9968	1.6522	1.3542	−0.0021	0.9998	0.0050	−98.54
	PMMA	1.6571	0.9828	0.0413	−48.44	1.8220	1.3223	0.9988	0.0108	−81.18	1.5741	1.3223	0.9988	0.0108	−81.18	1.7370	1.0555	0.9861	0.0372	−43.48	0.9885	1.8189	1.3479	0.0007	0.9989	0.0103	−78.46

## References

[1] Esper A., Mühlbauer W. (1998). Solar drying—An effective means of food preservation. Renew. Energy.

[2] Müller J., Mühlbauer W., Tsotsas E., Mujumdar A.S. (2012). Solar Drying. Modern Drying Technology.

[3] Udomkun P., Romuli S., Schock S., Mahayothee B., Sartas M., Wossen T., Njukwe E., Vanlauwe B., Müller J. (2020). Review of solar dryers for agricultural products in Asia and Africa: An innovation landscape approach. J. Environ. Manag..

[4] Prasad J., Vijay V.K., Tiwari G.N., Sorayan V.P.S. (2006). Study on performance evaluation of hybrid drier for turmeric (*Curcuma longa* L.) drying at village scale. J. Food Eng..

[5] Ssemwanga M., Makule E., Kayondo S.I. (2020). Performance analysis of an improved solar dryer integrated with multiple metallic solar concentrators for drying fruits. Sol. Energy.

[6] Janjai S., Lamlert N., Intawee P., Mahayothee B., Boonrod Y., Haewsungcharern M., Bala B.K., Nagle M., Müller J. (2009). Solar drying of peeled longan using a side loading type solar tunnel dryer: Experimental and simulated performance. Dry. Technol..

[7] Kumar A., Singh R., Prakash O., Ashutosh (2014). Review on global solar drying status. Agric. Eng. Int. CIGR J..

[8] Janjai S., Khamvongsa V., Bala B.K. (2007). Development, design, and performance of a PV-ventilated greenhouse dryer. Int. Energy J..

[9] Nabnean S., Nimnuan P. (2020). Case studies in thermal engineering experimental performance of direct forced convection household solar dryer for drying banana. Case Stud. Therm. Eng..

[10] Singh S., Kumar S. (2012). Testing method for thermal performance based rating of various solar dryer designs. Sol. Energy.

[11] Condorí M., Echazú R., Saravia L. (2001). Solar drying of sweet pepper and garlic using the tunnel greenhouse drier. Renew. Energy.

[12] Rodríguez-Ramírez J., Mendez-Lagunas L.L., Ĺopez-Ortiz A., Muñiz-Becerá S. (2021). Nair, K. Solar drying of strawberry using polycarbonate with UV protection and polyethylene covers: Influence on anthocyanin and total phenolic content. Sol. Energy.

[13] Elkhadraoui A., Kooli S., Hamdi I., Farhat A. (2015). Experimental investigation and economic evaluation of a new mixed-mode solar greenhouse dryer for drying of red pepper and grape. Renew. Energy.

[14] Janjai S., Intawee P., Kaewkiew J., Sritus C., Khamvongsa V. (2011). A large-scale solar greenhouse dryer using polycarbonate cover: Modeling and testing in a tropical environment of Lao People’s Democratic Republic. Renew. Energy.

[15] Janjai S., Keawprasert T. (2006). Design and performance of a solar tunnel dryer with a polycarbonate cover. Int. Energy J..

[16] Serrano M.A., Moreno J.C. (2020). Spectral transmission of solar radiation by plastic and glass materials. J. Photochem. Photobiol. B Biol..

[17] Janjai S. (2012). A greenhouse type solar dryer for small-scale dried food industries: Development and dissemination. Int. J. Energy Environ..

[18] Devan P.K., Bibin C., Asburris Shabrin I., Gokulnath R., Karthick D. (2020). Solar drying of fruits—A comprehensive review. Mater. Today Proc..

[19] Mohammed S., Edna M., Siraj K. (2020). The effect of traditional and improved solar drying methods on the sensory quality and nutritional composition of fruits: A case of mangoes and pineapples. Heliyon.

[20] Nelson K.M., Dahlin J.L., Bisson J., Graham J., Pauli G.F., Walters M.A. (2017). The essential medicinal chemistry of curcumin. J. Med. Chem..

[21] Sandur S.K., Pandey M.K., Sung B., Ahn K.S., Murakami A., Sethi G., Limtrakul P., Badmaev V., Aggarwal B.B. (2007). Curcumin, demethoxycurcumin, bisdemethoxycurcumin, tetrahydrocurcumin and turmerones differentially regulate anti-inflammatory and anti-proliferative responses through a ROS-independent mechanism. J. Carcinog..

[22] Suksamrarn A., Ponglikitmongkol M., Wongkrajang K., Chindaduang A., Kittidanairak S., Jankam A., Yingyongnarongkul B.E., Kittipanumat N., Chokchaisiri R., Khetkam P. (2008). Diarylheptanoids, new phytoestrogens from the rhizomes of *Curcuma comosa*: Isolation, chemical modification and estrogenic activity evaluation. Bioorg. Med. Chem..

[23] Mondal S., Ghosh S., Moulik S.P. (2016). Biology stability of curcumin in different solvent and solution media: UV—visible and steady-state fluorescence spectral study. JPB.

[24] Tønnensen H.H., Karlsen J., van Henegouwen G.B. (1986). Studies on curcumin and curcuminoids. Z. Lebensm. Unters. Forsch..

[25] Lee B.H., Choi H.A., Kim M.R., Hong J. (2013). Changes in chemical stability and bioactivities of curcumin by ultraviolet radiation. Food Sci. Biotechnol..

[26] Heger M., Van Golen R.F., Broekgaarden M., Michel M.C. (2014). The molecular basis for the pharmacokinetics and pharmacodynamics of curcumin and its metabolites in relation to cancers. Pharmacol. Rev..

[27] Raza A., Ali M.A., Yusof Y.A., Nasir A., Muneer S. (2018). Effect of different drying treatments on concentration of curcumin in raw *Curcuma longa* L. Food Res..

[28] Mahayothee B., Thamsala T., Khuwijitjaru P., Janjai S. (2020). Effect of drying temperature and drying method on drying rate and bioactive compounds in cassumunar ginger (*Zingiber montanum*). J. Appl. Res. Med. Aromat. Plants.

[29] Argyropoulos D., Heindl A., Müller J. (2011). Assessment of convection, hot-air combined with microwave-vacuum and freeze-drying methods for mushrooms with regard to product quality. Int. J. Food Sci. Technol..

[30] FAO (2004). Turmeric: Post-Harvest Operations.

[31] Crank J. (1975). The Mathematics of Diffusion.

[32] Özdemir M., Onur Devres Y. (1999). Thin layer drying characteristics of hazelnuts during roasting. J. Food Eng..

[33] Lewis W.K. (1921). The rate of drying of solid materials. J. Ind. Eng. Chem..

[34] Page G.E. (1949). Factors influencing the maximum rates of air drying shelled corn in thin layers. Master’s Thesis.

[35] Overhults D.G., White G.M., Hamilton H.E., Ross I.J. (1973). Drying soybeans with heated air. Trans. ASAE.

[36] Midilli A., Kucuk H. (2003). Mathematical modeling of thin layer drying of pistachio by using solar energy. Energy Convers. Manag..

[37] Akaike H. (1974). A new look at the statistical model identification. IEEE Trans. Automat. Contr..

[38] R Development Core Team (2008). R: A Language and Environment for Statistical Computing.

[39] Jödicke K., Arendt S., Hofacker W., Speckle W. (2020). The influence of process parameters on the quality of dried agricultural products determined using the cumulated thermal load. Dry. Technol..

[40] Ibrahim D., Zamfirescu C. (2016). Drying Phenomena: Theory and Applications.

[41] Jayas D.S., Cenkowski S., Pabis S., Muir W.E. (1991). Review of thin-layer drying and wetting equations review of thin-layer. Dry. Technol..

[42] Pal K., Chowdhury S., Dutta S.K., Chakraborty S., Chakraborty M., Pandit G.K., Dutta S., Paul P.K., Choudhury A., Majumder B. (2020). Analysis of rhizome colour content, bioactive compound profiling and *ex-situ* conservation of turmeric genotypes (*Curcuma longa* L.) from sub-Himalayan terai region of India. Ind. Crops Prod..

[43] Wichitnithad W., Jongaroonngamsang N., Pummangura S., Rojsitthisak P. (2009). A simple isocratic HPLC method for the simultaneous determination of curcuminoids in commercial turmeric extracts. Phytochem. Anal..

[44] Komonsing N., Khuwijitjaru P., Nagle M., Müller J., Mahayothee B. (2022). Effect of drying temperature together with light on drying characteristics and bioactive compounds in turmeric slice. J. Food Eng..

[45] Priyadarsini K.I. (2009). Photophysics, photochemistry and photobiology of curcumin: Studies from organic solutions, bio-mimetics and living cells. J. Photochem. Photobiol. C Photochem. Rev..

[46] Chumroenphat T., Somboonwatthanakul I., Saensouk S., Siriamornpun S. (2021). Changes in curcuminoids and chemical components of turmeric (*Curcuma longa* L.) under freeze-drying and low-temperature drying methods. Food Chem..

[47] Souza C.R.A., Osme S.F., Glória M.B.A. (1997). Stability of curcuminoid pigments in model systems. J. Food Process. Preserv..

[48] Suyitno S., Maret U.S., Kristiawan B., Maret U.S., Wibowo A.H., Maret U.S. (2018). Effect of light and temperature on the efficiency and stability of curcumin- dye-sensitized solar cells. Int. Energy J..

[49] Tønnesen H.H., Karlsen J. (1985). Studies on curcumin and curcuminoids—V. Alkaline Degradation of Curcumin. Z. Lebensm. Unters. Forsch..

